# Prognostic Nomograms for Patients With NF‐Pan‐NET After Pancreatectomy: A Retrospective Analysis Based on SEER Database

**DOI:** 10.1002/cnr2.2165

**Published:** 2024-09-05

**Authors:** Yizhi Wang, Yang Kong, Qifan Yang, Dongkai Zhou

**Affiliations:** ^1^ Department of Hepatobiliary and Pancreatic Surgery, The Second Affiliated Hospital Zhejiang University School of Medicine Hangzhou China; ^2^ Key Laboratory of Precision Diagnosis and Treatment for Hepatobiliary and Pancreatic Tumor of Zhejiang Province Hangzhou China; ^3^ Research Center of Diagnosis and Treatment Technology for Hepatocellular Carcinoma of Zhejiang Province Hangzhou China; ^4^ Clinical Medicine Innovation Center of Precision Diagnosis and Treatment for Hepatobiliary and Pancreatic Disease of Zhejiang University Hangzhou China; ^5^ Clinical Research Center of Hepatobiliary and Pancreatic Diseases of Zhejiang Province Hangzhou China; ^6^ Cancer Center Zhejiang University Hangzhou China

**Keywords:** nomogram, pancreatectomy, pancreatic neuroendocrine tumors, prognosis, SEER

## Abstract

**Aims:**

Surgical resection is the primary treatment option for patients diagnosed with nonfunctional pancreatic neuroendocrine tumors (NF‐Pan‐NETs). However, the postoperative prognostic evaluation for NF‐Pan‐NET patients remains obscure. This study aimed to construct an efficient model to predict the prognosis of NF‐Pan‐NET patients who have received surgical resection.

**Methods:**

NF‐Pan‐NET patients after pancreatectomy were retrieved from the SEER database for the period of 2010 to 2019. A total of 2844 patients with NF‐Pan‐NET from SEER database were included in our study. After careful screening, six clinicopathological variables including age, grade, AJCC T stage, AJCC N stage, AJCC M stage, and chemotherapy were selected to develop nomograms to predict overall survival (OS) and cancer‐specific survival (CSS) respectively of the patients.

**Results:**

The novel models demonstrated high accuracy and discrimination in prognosticating resected NF‐Pan‐NET through various validation methods. Furthermore, the risk subgroups classified by the newly developed risk stratification systems based on the nomograms exhibited significant differences in both OS and CSS, surpassing the efficacy of the AJCC 8th TNM staging system. Novel nomograms and corresponding risk classification systems were developed to predict OS and CSS in patients with NF‐Pan‐NET after pancreatectomy.

**Conclusion:**

The models demonstrated superior performance compared to traditional staging systems, providing clinicians with more accurate and personalized guidance for postoperative surveillance and treatment.

## 1 Introduction

Pancreatic neuroendocrine tumors (Pan‐NET) are a heterogeneous group of tumors originating from diverse pancreatic neuroendocrine cells that can be roughly classified as functional and nonfunctional Pan‐NET (NF‐Pan‐NET) based on the types of the secreted hormones [1]. Although Pan‐NET is relatively rare pancreatic tumor type compared to other pancreatic tumor types, its morbidity has been dramatically increasing nowadays due to improved early detection methods and the widespread adoption of routine physical examination [2, 3]. It is estimated that NF‐Pan‐NET accounts for approximately 60%–90% of all Pan‐NETs [4]. Despite recent advancements in comprehensive therapeutics, surgical resection remains the only curative treatment option for NF‐Pan‐NET management [5]. Considering the malignant potential of NF‐Pan‐NET, several studies have reported survival benefits associated with surgical resection not only in patients with small (<2 cm) tumors but also those with locally advanced tumors or even distant metastases [6, 7]. Therefore, according to the latest European Neuroendocrine Tumor Society (ENETS) guideline, personalized management strategies should be employed for patients with NF‐Pan‐NET measuring >1 cm and ≤2 cm based on both surgical modality and patient comorbidity considerations [8]. Moreover, surgical resection can also enhance the efficacy of adjuvant therapy in Pan‐NET patients [9].

Although a majority of NF‐Pan‐NET patients undergo pancreatectomy, the recognized prognostic evaluation system for these patients is still lacking. The traditional American Joint Committee on Cancer (AJCC) tumor–node–metastasis (TNM) staging systems are well‐constructed and the 8th version exhibited improved efficacy in prognostic prediction [10, 11]. In addition, tumor grade based on Ki‐67 proliferative index and mitotic rate is also a crucial clinicopathological parameter for determining survival among different risk subgroups of Pan‐NET patients [12]. Therefore, combining tumor grade with the AJCC TNM staging system showed greater potential in predicting prognosis and guiding chemotherapy selection for NF‐Pan‐NET patients [13]. It has been reported that incorporating tumor grade improves the prognostic prediction efficacy of the AJCC TNM staging system in terms of cancer‐specific survival (CSS) among Pan‐NET patients [14]. However, consensus has not yet been reached regarding prognostic factors and risk stratification systems for predicting survival in NF‐Pan‐NET patients after pancreatectomy, which is meaningful and important for both patients and clinicians.

In this study, we developed user‐friendly nomograms for predicting both overall survival (OS) and CSS in patients with NF‐Pan‐NET after pancreatectomy using key clinicopathological variables derived from the Surveillance, Epidemiology, and End Results (SEER) database. Various validation methods were recruited to prove the accuracy, clinical applicability and feasibility of the novel nomograms. Ultimately, we established novel risk stratification systems for NF‐Pan‐NET based on these nomograms.

## 2 Methods

### 2.1 Patient Selection

The original data of this population‐based study were obtained from SEER database using SEER*Stat software Version 8.4.2 (http://seer.cancer.gov/seerstat/) [15]. We utilized this version of the SEER*Stat Database: Incidence‐SEER Research Plus Data, 17 Registries, Nov 2021 Sub (2020–2019) to retrieve the data for our study. Patients included in this analysis were selected based on primary site codes C25.0 to C25.9, and NF‐Pan‐NETs were defined according to International Classification of Disease for Oncology 3rd edition (ICD‐O‐3) site record, including pancreatic endocrine tumor (8150), carcinoid tumor (8240), enterochromaffin cell carcinoid (8241), enterochromaffin‐like cell tumor (8242), goblet cell carcinoid (8243), mixed adenoneuroendocrine carcinoma (8244), adenocarcinoid tumor (8245), neuroendocrine carcinoma (8246), and atypical carcinoid tumor (8249). As per regulations regarding public access and anonymity of SEER data, this study was exempted by the Institutional Review Board of Second Affiliated Hospital of Zhejiang University.

**FIGURE 1:**
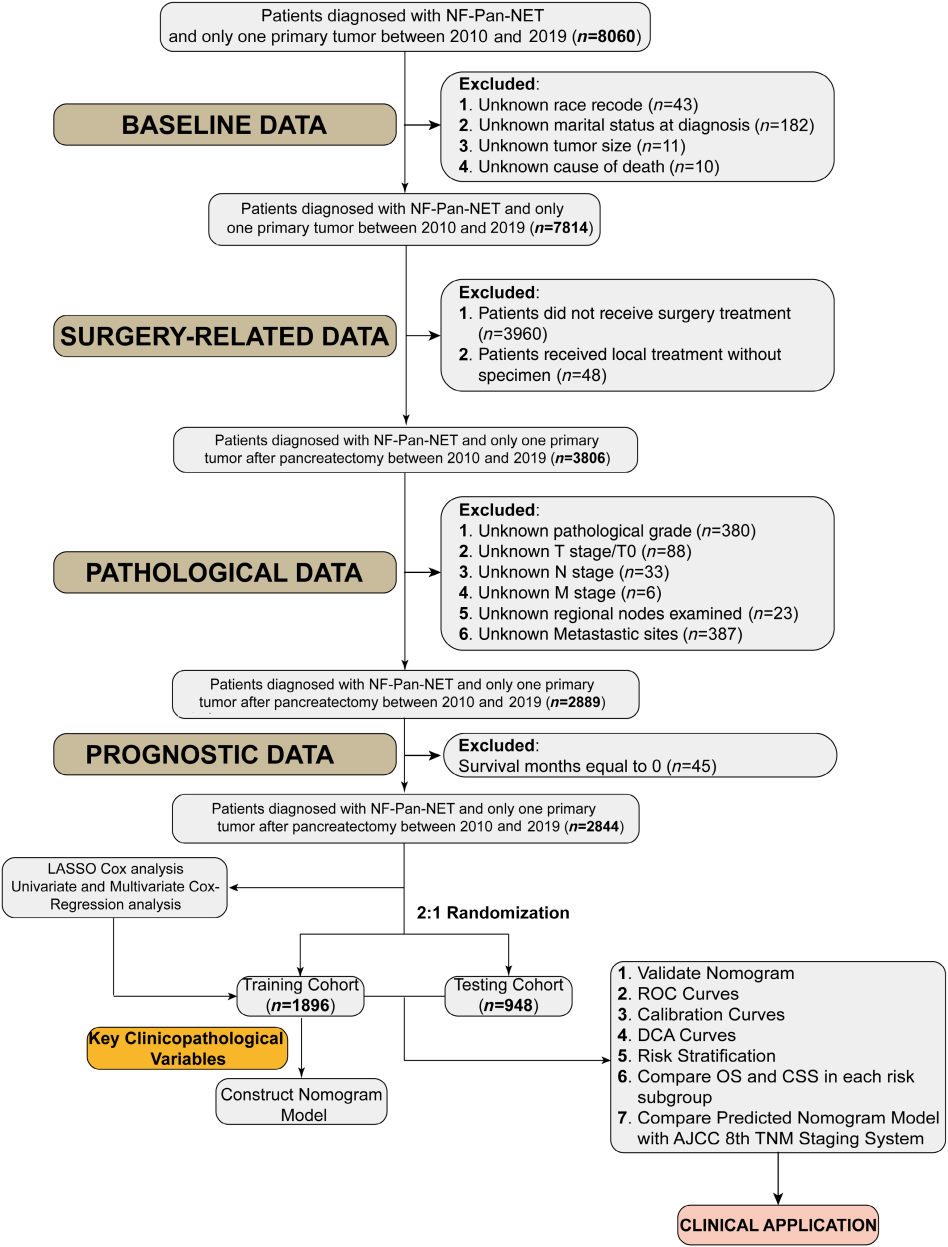
Flow diagram of NF‐Pan‐NET patient's inclusion in SEER database.

The inclusion criteria were as follows: (1) pathological diagnosis of NF‐Pan‐NET, (2) pancreatectomy conducted, and (3) exclusion of other malignancies. The exclusion criteria were also as follows: (1) blank/NA information; (2) absence of pancreatectomy; and (3) absence of essential clinicopathological variables, including race, marital status, grade, stage, tumor size, regional nodes examined (RNE), distant metastases status, causes of death (COD), and survival months. The specific screening flow diagram is presented in Figure 1. Supplementary file 1 provides detailed information on the included patients from the SEER database.

### 2.2 Data Collection and Variable Definition

For each patient, the following fields from SEER database were retrieved: Age, Gender, Year of diagnosis, Race, Primary site, Grade, RX Summ—Surg/Rad Seq, RX Summ—Systemic/Sur Seq, Chemotherapy recode (yes, no/unk), Derived EOD 2018 T (2018+), Derived EOD 2018 N (2018+), Derived EOD 2018 M (2018+), Derived EOD 2018 Stage Group (2018+), Derived AJCC T, 7th ed. (2010–2015), Derived AJCC N, 7th ed. (2010–2015), Derived AJCC M, 7th ed. (2010–2015), Derived AJCC Stage Group, 7th ed. (2010–2015), Derived SEER Combined T (2016–2017), Derived SEER Combined N (2016–2017), Derived SEER Combined M (2016–2017), Derived SEER Cmb Stg Grp (2016–2017), Derived AJCC T, 6th ed. (2004–2015), Derived AJCC N, 6th ed. (2004–2015), CS mets at dx (2004–2015), Derived AJCC M, 6th ed. (2004–2015), Derived AJCC Stage Group, 6th ed. (2004–2015), EOD Primary Tumor (2018+), EOD Regional Nodes (2018+), EOD Mets (2018+), CS lymph nodes (2004–2015), CS extension (2004–2015), SEER Combined Mets at DX‐bone (2010+), SEER Combined Mets at DX‐brain (2010+), SEER Combined Mets at DX‐liver (2010+), SEER Combined Mets at DX‐lung (2010+), Mets at DX‐Distant LN (2016+), Mets at DX‐Other (2016+), RNE (1988+), Regional nodes positive (1988+), Marital status at diagnosis, COD to site code and survival information. These fields were collected for updating AJCC TNM stage into AJCC 8th TNM staging system to enhance statistical accuracy.

The SEER database included both pathological and clinical T stage, without distinguishing between them in our study. Histologic grade information was utilized to categorize the tumor into different grades: G1 (well differentiated), G2 (moderately differentiated), G3 (poorly differentiated), and G4 (undifferentiated or anaplastic). Due to a relatively small number of cases in the G4 category, they were combined with G3 for subsequent analyses. The primary outcome was OS and while CSS served as the secondary outcome measure. OS was defined as the time from diagnosis until death or last follow‐up, whereas CSS referred to the time from diagnosis until death specifically caused by NF‐Pan‐NET or last follow‐up. Optimal cutoff points for age, tumor size, number of RNE, and year of diagnosis were determined using X‐tile software from Yale School of Medicine, CT, USA [16] (Figure S1).

### 2.3 Statistical Analysis

Statistical analyses were performed using SPSS statistics (version 27.0, Armonk, NY, USA) to identify predictors, while R software (version 4.3.1; https://www.r‐project.org) was utilized to generate the statistical diagram. Categorical variables were compared using the Chi‐square test or Fisher's exact test. Cox regression analysis incorporating LASSO, univariate, and multivariate approaches was conducted to identify key clinicopathological variables associated with hazard ratios (HRs) and corresponding 95% confidence intervals (CIs). A significance level of two‐tailed *p* < 0.05 was considered statistically significant for all tests.

The included patients were randomly divided into a training cohort and a testing cohort in a nearly 2:1 ratio. The training cohort was utilized to develop prognostic nomograms for OS and CSS based on the screened key clinicopathological variables, while the testing cohort was employed to validate the performance of the models using Harrell's concordance index (C‐index), area under the curve (AUC) of receiver‐operating characteristic curves (ROC), calibration curve, decision curve analyses (DCA), and clinical impact curve (CIC). The predictive accuracy of the nomograms was evaluated through calculation of AUC and C‐index. Calibration curves were used to assess consistency between predicted and actual values obtained from the nomograms. DCA and CIC were applied to evaluate the clinical applicability of the nomograms, comparing their predictive efficacy with that of AJCC 8th TNM staging system. Risk scores of all patients in OS and CSS from the SEER database according to the nomograms were also calculated, followed by stratification into low‐, medium‐, and high‐risk subgroups within both training and testing cohorts using optimal cutoff values determined by X‐tile software. Finally, Kaplan–Meier curves with the log‐rank test were conducted to compare the OS and CSS among different risk subgroups.

## 3 Results

### 3.1 Baseline Characteristic of the Patients

Two thousand eight hundred forty‐four eligible NF‐Pan‐NET patients after pancreatectomy from SEER database were included. Among them, 1896 patients were assigned to the training cohort. while 948 patients were assigned to the testing cohort. In the overall patient cohort, the median age at diagnosis was 58 (7–94) years, and there was no significant difference in morbidity between male and female. The majority of the patients were the white (77.4%) and married (66.2%). G1 was found to be the predominant pathological grade (73.8%). The median tumor size for resected NF‐Pan‐NET was 25 mm with T3 stage being most common (35%). Only a small proportion of patients after pancreatectomy were found positive metastatic lymph nodes (24.9%). Based on X‐tile analysis, age at diagnosis was categorized into three groups using optimal cutoff points “58” and “71”: <58 years, 58–71 years, and > 71 years. Similarly, tumor size was divided into <25 mm, 25–35 mm, and > 35 mm groups, while the number of RNE was classified into 0–1, 2–14 and >14 groups. Table 1 presents baseline clinicopathological variables and treatment strategies for both training and testing cohorts. All features were comparable between the two cohorts except for age at diagnosis and race.

**TABLE 1:** Baseline characteristics of NF‐Pan‐NET patients after pancreatectomy in the total, the training, and testing cohort.

Variables	Total cohort (*n* = 2844)	Training cohort (*n* = 1896)	Testing cohort (*n* = 948)	*p* ^a^
Age, years, *n* (%)^c^				**0.002**
<58	1331 (46.8)	856 (45.1)	475 (50.1)	
58–71	1146 (40.3)	807 (42.6)	339 (35.8)	
>71	367 (12.9)	233 (12.3)	134 (14.1)	
Gender, *n* (%)				0.596
Female	1376 (48.4)	924 (48.7)	452 (47.7)	
Male	1468 (51.6)	972 (51.3)	496 (52.3)	
Race, *n* (%)				**0.021**
Other	328 (11.5)	239 (12.6)	89 (9.4)	
Black	314 (11.1)	198 (10.4)	116 (12.2)	
White	2202 (77.4)	1459 (77.0)	743 (78.4)	
Year of diagnosis, *n* (%)				0.876
2010–2012	539 (19.0)	355 (18.7)	184 (19.4)	
2013–2015	905 (31.8)	602 (31.8)	303 (32.0)	
2016–2019	1400 (49.2)	939 (49.5)	461 (48.6)	
Marital status, *n* (%)				0.906
Other	448 (15.8)	298 (15.7)	150 (15.8)	
Married	1883 (66.2)	1260 (66.5)	623 (65.7)	
Single	513 (18.0)	338 (17.8)	175 (18.5)	
Primary site, *n* (%)				0.898
Other	406 (14.3)	273 (14.4)	133 (14.0)	
Head	776 (27.3)	509 (26.8)	267 (28.2)	
Body	475 (16.7)	320 (16.9)	155 (16.3)	
Tail	1187 (41.7)	794 (41.9)	393 (41.5)	
Grade, *n* (%)				0.547
G1	2099 (73.8)	1411 (74.4)	688 (72.6)	
G2	626 (22.0)	406 (21.4)	220 (23.2)	
G3	119 (4.2)	79 (4.2)	40 (4.2)	
AJCC T stage, *n* (%)				0.109
T1	953 (33.5)	619 (32.6)	334 (35.2)	
T2	826 (29.0)	561 (29.6)	265 (28.0)	
T3	994 (35.0)	676 (35.7)	318 (33.5)	
T4	71 (2.5)	40 (2.1)	31 (3.3)	
Tumor size, mm, *n* (%)^c^				0.251
<25	1340 (47.1)	875 (46.1)	465 (49.1)	
25–35	598 (21.0)	413 (21.8)	185 (19.5)	
>35	906 (31.9)	608 (32.1)	298 (31.4)	
AJCC N stage, *n* (%)				0.408
N0	2136 (75.1)	1433 (75.6)	703 (74.2)	
N1	708 (24.9)	463 (24.4)	245 (25.8)	
RNE, *n* (%)^c^				0.831
0–1	637 (22.4)	431 (22.7)	206 (21.7)	
2–14	1350 (47.5)	895 (47.2)	455 (48.0)	
>14	857 (30.1)	570 (30.1)	287 (30.3)	
AJCC M stage, *n* (%)				0.389
M0	2575 (90.5)	1723 (90.9)	852 (89.9)	
M1	269 (9.5)	173 (9.1)	96 (10.1)	
Bone metastasis, *n* (%)				0.453^b^
No	2836 (99.7)	1892 (99.8)	944 (99.6)	
Yes	8 (0.3)	4 (0.2)	4 (0.4)	
Brain metastasis, *n* (%)				1^b^
No	2843 (99.9)	1895 (99.9)	948 (100.0)	
Yes	1 (0.1)	1 (0.1)	0 (0.0)	
Liver metastasis, *n* (%)				1^b^
No	2601 (91.5)	1734 (91.5)	867 (91.5)	
Yes	243 (8.5)	162 (8.5)	81 (8.5)	
Lung metastasis, *n* (%)				0.231^b^
No	2837 (99.8)	1893 (99.8)	944 (99.6)	
Yes	7 (0.2)	3 (0.2)	4 (0.4)	
Surgical modality, *n* (%)				0.443
Local resection	35 (1.2)	25 (1.3)	10 (1.0)	
Partial pancreatectomy	187 (6.6)	120 (6.3)	67 (7.1)	
Pancreaticoduodenectomy	1351 (47.5)	920 (48.6)	431 (45.5)	
Total pancreatectomy	1039 (36.5)	674 (35.5)	365 (38.5)	
Pancreatectomy NOS	232 (8.2)	157 (8.3)	75 (7.9)	
Chemotherapy, *n* (%)				0.860
No	2691 (94.6)	1795 (94.7)	896 (94.5)	
Yes	153 (5.4)	101 (5.3)	52 (5.5)	
Radiotherapy, *n* (%)				0.599
No	2798 (98.1)	1867 (98.5)	931 (98.2)	
Yes	46 (1.9)	29 (1.5)	17 (1.8)	

*Note:* Bold values indicate that *p* < 0.05 which are statistically significant.

Abbreviations: AJCC, American Joint Committee on Cancer; NF‐Pan‐NET, nonfunctional pancreatic neuroendocrine tumor; NOS, not otherwise specified; RNE, regional nodes examined.

^a^ Chi‐square tests.

^b^ Fisher's exact test.

^c^ The cutoff values of continuous variables were determined via X‐tile software.

The median follow‐up duration for the patients was 41 months (range 1–119 months). During this observation period, a total of 277 patients (9.7%) succumbed to all causes, while there were 189 cancer‐specific deaths (6.6%). The cumulative survival of the entire cohort is presented in Table 2. The cumulative CSSs consistently surpassed the corresponding OSs at all time points.

**TABLE 2:** Cumulative survival of the entire cohort in our study.

	Cumulative OS	Cumulative CSS
1‐year survival (%)	97.0	97.8
3‐year survival (%)	93.2	94.9
5‐year survival (%)	89.1	92.4
7‐year survival (%)	83.6	88.9
9‐year survival (%)	77.4	84.3

### 3.2 Nomograms Establishment

**FIGURE 2:**
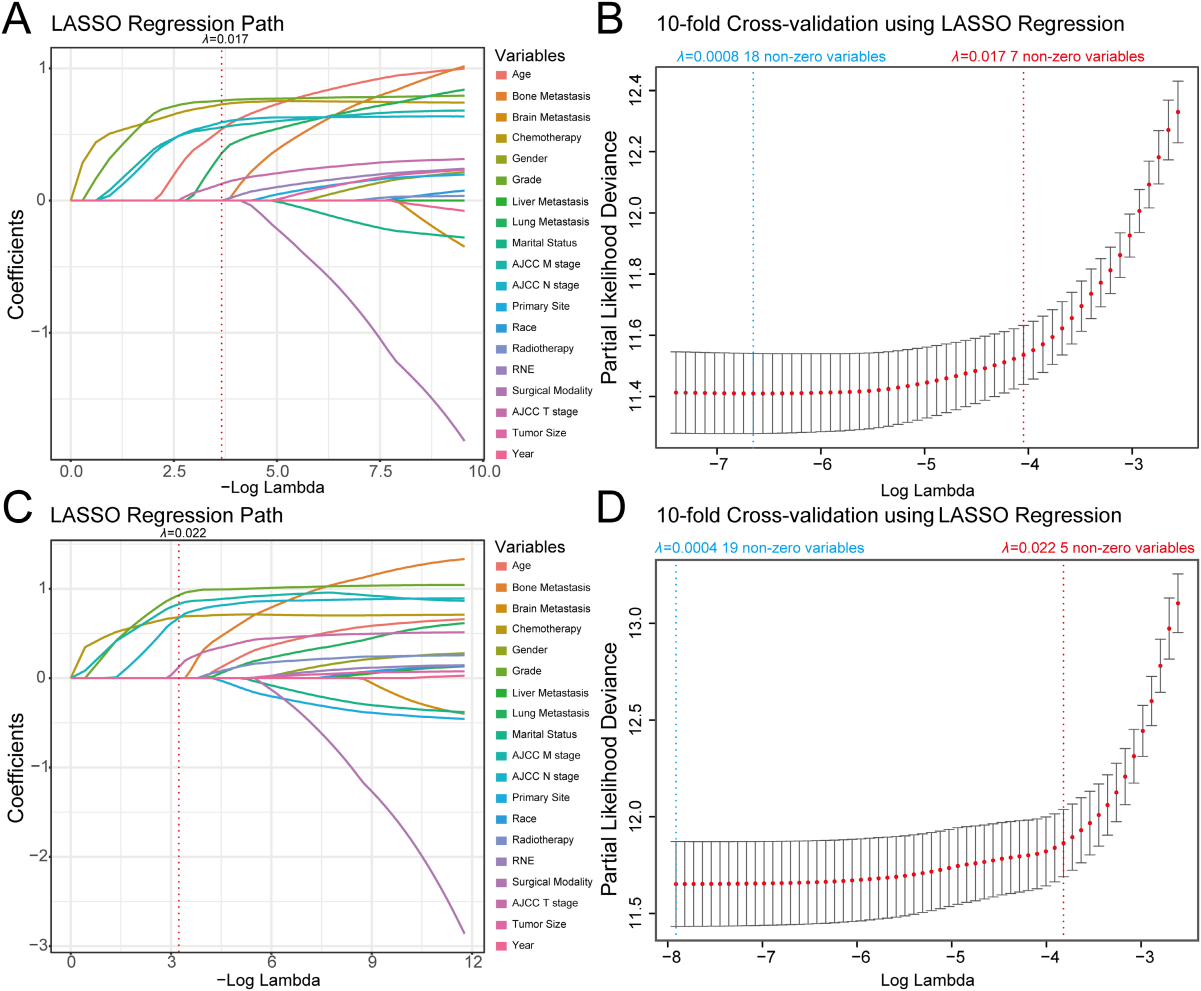
Plot of LASSO coefficient profiles of the 19 candidate predictors for OS (A) and CSS (C). Plot of partial likelihood deviance for OS (B) and CSS (D); the left vertical dotted lines were drawn at the values of log (l) chosen by minimum criteria, and the right vertical dotted lines were drawn at the values of log (l) chosen by one standard error of the minimum criteria.

**FIGURE 3:**
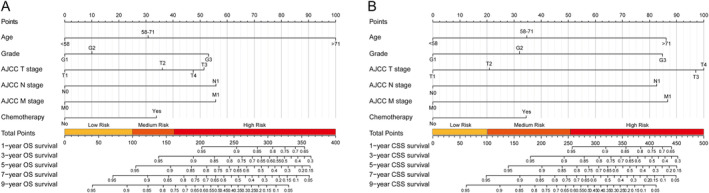
Nomograms for predicting the OS (A) and CSS (B) of NF‐Pan‐NET patients after pancreatectomy. The nomogram was established in the training cohort based on LASSO and multivariable COX regression, consisting of age, grade, AJCC T stage, AJCC N stage, AJCC M stage, and chemotherapy.

To identify the key clinicopathological variables for constructing nomograms, we initially conducted LASSO Cox analysis. The result revealed that out of the 19 included clinicopathological variables, 7 (chemotherapy, grade, AJCC M stage, AJCC N stage, age at diagnosis, lung metastasis, and AJCC T stage) and 5 (chemotherapy, AJCC M stage, grade, AJCC N stage, and AJCC T stage) nonzero variables were selected for OS and CSS prediction, respectively (Figure 2). Subsequently, univariate and multivariate Cox regression analyses were conducted to determine the independent prognostic factors associated with OS and CSS in these patients. The findings demonstrated that OS was significantly influenced by age at diagnosis, marital status, grade level, AJCC N stage, surgical modality as well as chemotherapy, whereas CSS was found to be associated with age at diagnosis, marital status, primary site, grade, AJCC N stage, AJCC T stage, AJCC M stage, and chemotherapy in multivariate analysis (Tables 3 and 4). According to the results of LASSO as well as univariate and multivariate Cox regression analyses, nomograms predicting 1‐, 3‐, 5‐, 7‐, and 9‐year OS as well as CSS were developed based on six clinicopathological variables including AJCC T stage, AJCC N stage, AJCC M stage, age at diagnosis, grade, and chemotherapy (Figure 3). To utilize the nomograms, patients can determine the score for each predictor by drawing a vertical line intersecting with the “Points” reference line. Subsequently, the cumulative scores can be identified on the “Total Points” line, and a perpendicular line is drawn downwards to intersect with the survival lines in order to obtain 1‐, 3‐, 5‐, 7‐, and 9‐year survival rates. For ease of calculation, the detailed score for each clinicopathological variable included in the nomograms predicting OS and CSS of NF‐Pan‐NET patients after pancreatectomy was exhibited in Table 5.

**TABLE 3:** Univariate and multivariate Cox regression for analyzing prognostic factors of OS in NF‐Pan‐NET patients after pancreatectomy.

		Univariate analysis			Multivariate analysis		
Variables	Number	HR	95% CI	*p* ^a^	HR	95% CI	*p* ^a^
Age, years^b^				**<0.001**			**<0.001**
<58	1331	Ref			Ref		
58–71	1146	1.665	1.268–2.185	**<0.001**	1.683	1.256–2.238	**<0.001**
>71	367	3.039	2.204–4.189	**<0.001**	3.588	2.541–5.065	**<0.001**
Gender				0.126			
Female	1376	Ref					
Male	1468	1.204	0.949–1.527	0.126			
Race				0.560			
Other	328	Ref					
Black	314	1.262	0.765–2.106	0.373			
White	2202	1.240	0.831–1.850	0.292			
Year of diagnosis				**0.036**			0.660
2010–2012	539	Ref			Ref		
2013–2015	905	0.757	0.571–1.005	0.054	0.958	0.714–1.284	0.773
2016–2019	1400	0.646	0.451–0.925	**0.017**	0.842	0.580–1.223	0.368
Marital status				**0.025**			**0.044**
Other	448	Ref			Ref		
Married	1883	0.665	0.496–0.893	**0.007**	0.710	0.523–0.964	**0.028**
Single	513	0.729	0.498–1.068	0.105	0.937	0.621–1.412	0.754
Primary site				**0.001**			0.215
Other	406	Ref			Ref		
Head	776	1.266	0.890–1.802	0.189	1.122	0.753–1.671	0.571
Body	475	0.645	0.409–1.017	0.059	0.790	0.494–1.261	0.323
Tail	1187	0.808	0.565–1.154	0.24	0.798	0.553–1.152	0.229
Grade				**<0.001**			**<0.001**
G1	2099	Ref			Ref		
G2	626	1.735	1.305–2.307	**<0.001**	1.371	1.016–1.850	**0.039**
G3	119	6.931	5.053–9.508	**<0.001**	2.234	1.514–3.296	**<0.001**
AJCC T stage				**<0.001**			0.360
T1	953	Ref			Ref		
T2	826	1.949	1.297–2.930	**0.001**	1.222	0.727–2.057	0.449
T3	994	3.960	2.774–5.654	**<0.001**	1.438	0.825–2.504	0.200
T4	71	4.555	2.197–9.442	**<0.001**	0.925	0.380–2.254	0.864
Tumor size, mm^b^				**<0.001**			0.423
<25	1340	Ref			Ref		
25–35	598	2.456	1.765–3.417	**<0.001**	1.300	0.834–2.026	0.246
>35	906	3.038	2.263–4.079	**<0.001**	1.100	0.695–1.742	0.684
AJCC N stage				**<0.001**			**<0.001**
N0	2136	Ref			Ref		
N1	708	3.507	2.769–4.441	**<0.001**	1.830	1.381–2.425	**<0.001**
RNE^b^				**<0.001**			0.190
0–1	637	Ref			Ref		
2–14	1350	2.031	1.381–2.987	**<0.001**	1.125	0.747–1.695	0.572
>14	857	3	2.024–4.447	**<0.001**	1.399	0.901–2.173	0.135
AJCC M stage				**<0.001**			0.066
M0	2575	Ref			Ref		
M1	269	4.130	3.187–5.351	**<0.001**	2.041	0.953–4.371	0.066
Bone metastasis				**<0.001**			0.191
No	2836	Ref			Ref		
Yes	8	6.276	2.336–16.864	**<0.001**	2.250	0.668–7.576	0.191
Brain metastasis				**<0.001**			0.902
No	2843	Ref			Ref		
Yes	1	37.084	5.153–266.869	**<0.001**	0.845	0.058–12.244	0.902
Liver metastasis				**<0.001**			0.969
No	2601	Ref			Ref		
Yes	243	3.862	2.948–5.058	**<0.001**	0.985	0.454–2.137	0.969
Lung metastasis				**<0.001**			0.289
No	2837	Ref			Ref		
Yes	7	7.401	3.052–17.945	**<0.001**	1.884	0.584–6.080	0.289
Surgical modality				**<0.001**			**0.036**
Pancreatectomy NOS	35	Ref			Ref		
Local resection	187	0.243	0.015–3.886	0.317	0.545	0.033–8.916	0.670
Partial pancreatectomy	1351	3.638	0.508–26.068	0.199	4.905	0.678–35.476	0.115
Pancreaticoduodenectomy	1039	5.592	0.781–40.015	0.086	4.303	0.592–31.254	0.149
Total pancreatectomy	232	6.610	0.904–48.352	0.063	6.231	0.843–46.044	0.073
Chemotherapy				**<0.001**			**<0.001**
No	2691	Ref			Ref		
Yes	153	5.522	4.176–7.303	**<0.001**	2.055	1.437–2.940	**<0.001**
Radiotherapy				**<0.001**			0.540
No	2798	Ref			Ref		
Yes	46	3.466	2.091–5.743	**<0.001**	1.188	0.684–2.064	0.540

*Note:* Bold values indicate that *p* < 0.05 which are statistically signifcant.

Abbreviations: AJCC, American Joint Committee on Cancer; CI, confidential interval; HR, hazard rate; NF‐Pan‐NET, nonfunctional pancreatic neuroendocrine tumor; NOS, not otherwise specified; OS, overall survival; RNE, regional nodes examined.

^a^ Cox proportional hazards model.

^b^ The cutoff values of continuous variables were determined via X‐tile software.

**TABLE 4:** Univariate and multivariate Cox regression for analyzing prognostic factors of CSS in NF‐Pan‐NET patients after pancreatectomy.

		Univariate analysis			Multivariate analysis		
Variables	Number	HR	95% CI	*p* ^a^	HR	95% CI	*p* ^a^
Age, years^b^				**0.003**			**0.002**
<58	1331	Ref			Ref		
58–71	1146	1.455	1.062–1.995	**0.020**	1.402	1.004–1.957	**0.047**
>71	367	1.972	1.304–2.982	**0.001**	2.201	1.415–3.424	**<0.001**
Gender				0.101			
Female	1376	Ref					
Male	1468	1.273	0.954–1.699	0.101			
Race				0.447			
Other	328	Ref					
Black	314	0.954	0.496–1.835	0.887			
White	2202	1.235	0.767–1.989	0.386			
Year of diagnosis				0.177			
2010–2012	539	Ref			Ref		
2013–2015	905	0.787	0.557–1.113	0.176			
2016–2019	1400	0.684	0.448–1.044	0.078			
Marital status				**0.010**			**0.011**
Other	448	Ref			Ref		
Married	1883	0.586	0.414–0.828	**0.002**	0.576	0.401–0.413	**0.003**
Single	513	0.659	0.419–1.037	0.071	0.676	0.413–1.106	0.119
Primary site				**<0.001**			**0.021**
Other	406	Ref			Ref		
Head	776	1.217	0.808–1.833	0.347	1.210	0.751–1.951	0.433
Body	475	0.615	0.361–1.048	0.074	0.938	0.539–1.632	0.820
Tail	1187	0.640	0.417–0.982	**0.041**	0.627	0.401–0.981	**0.041**
Grade				**<0.001**			**<0.001**
G1	2099	Ref			Ref		
G2	626	2.424	1.727–3.402	**<0.001**	1.714	1.203–2.442	**0.003**
G3	119	11.063	7.746–15.801	**<0.001**	3.298	2.128–5.111	**<0.001**
AJCC T stage				**<0.001**			0.205
T1	953	Ref			Ref		
T2	826	2.177	1.186–3.995	**0.012**	1.023	0.470–2.230	0.954
T3	994	7.277	4.332–12.224	**<0.001**	1.617	0.732–3.571	0.235
T4	71	10.469	4.623–23.708	**<0.001**	1.152	0.396–3.350	0.794
Tumor size, mm^b^				**<0.001**			0.442
<25	1340	Ref			Ref		
25–35	598	3.491	2.215–5.502	**<0.001**	1.503	0.803–2.810	0.203
>35	906	5.443	3.635–8.151	**<0.001**	1.357	0.727–2.535	0.338
AJCC N stage				**<0.001**			**<0.001**
N0	2136	Ref			Ref		
N1	708	5.638	4.184–7.599	**<0.001**	2.362	1.662–3.358	**<0.001**
RNE^b^				**<0.001**			0.734
0–1	637	Ref			Ref		
2–14	1350	2.352	1.435–3.855	**<0.001**	0.954	0.561–1.623	0.862
>14	857	3.519	2.131–5.812	**<0.001**	1.087	0.619–1.909	0.772
AJCC M stage				**<0.001**			**0.048**
M0	2575	Ref			Ref		
M1	269	6.704	5.011–8.967	**<0.001**	2.384	1.006–5.649	**0.048**
Bone metastasis				**<0.001**			0.073
No	2836	Ref			Ref		
Yes	8	9.180	3.403–24.762	**<0.001**	3.053	0.902–10.340	0.073
Brain metastasis				**<0.001**			0.987
No	2843	Ref			Ref		
Yes	1	50.557	6.992–365.585	**<0.001**	0.977	0.060–15.846	0.987
Liver metastasis				**<0.001**			0.770
No	2601	Ref			Ref		
Yes	243	6.275	4.661–8.447	**<0.001**	1.138	0.479–2.706	0.770
Lung metastasis				**<0.001**			0.501
No	2837	Ref			Ref		
Yes	7	8.709	3.230–23.479	**<0.001**	1.589	0.412–6.135	0.501
Surgical modality				**0.005**			0.294
Pancreatectomy NOS	35	Ref			Ref		
Local resection	187	0	0–7.988e^97	0.927	0	0–1.954e^98	0.934
Partial pancreatectomy	1351	2.337	0.325–16.830	0.399	3.519	0.482–25.703	0.215
Pancreaticoduodenectomy	1039	3.904	0.544–28.031	0.176	2.762	0.376–20.284	0.318
Total pancreatectomy	232	4.538	0.612–33.634	0.139	4.313	0.573–32.489	0.156
Chemotherapy				**<0.001**			**0.002**
No	2691	Ref			Ref		
Yes	153	7.897	5.787–10.775	**<0.001**	1.866	1.259–2.765	**0.002**
Radiotherapy				**<0.001**			0.190
No	2798	Ref			Ref		
Yes	46	5.231	3.133–8.733	**<0.001**	1.461	0.829–2.574	0.190

*Note:* Bold values indicate that *p* < 0.05 which are statistically signifcant.

Abbreviations: AJCC: American Joint Committee on Cancer; CI: confidential interval; CSS: cancer‐specific survival; HR: hazard rate; NF‐Pan‐NET: nonfunctional pancreatic neuroendocrine tumor; NOS: not otherwise specified; RNE: regional nodes examined.

^a^ Cox proportional hazards model.

^b^ The cutoff values of continuous variables were determined via X‐tile software.

**TABLE 5:** Score of each clinicopathological variable in our nomogram predicting OS and CSS of NF‐Pan‐NET patients after pancreatectomy.

Variables	Nomogram score of OS	Nomogram score of CSS
Age, years		
<58	0	0
58–71	31	35
>71	100	86
Grade		
G1	0	0
G2	10	32
G3	53	85
AJCC T stage		
T1	0	0
T2	36	21
T3	52	100
T4	47	97
AJCC N stage		
N0	0	0
N1	56	83
AJCC M stage		
M0	0	0
M1	56	87
Chemotherapy		
No	0	0
Yes	34	34

Abbreviations: AJCC: American Joint Committee on Cancer; CSS: cancer‐specific survival; NF‐Pan‐NET: nonfunctional pancreatic neuroendocrine tumor; OS: overall survival.

### 3.3 Nomograms Validation

**FIGURE 4:**
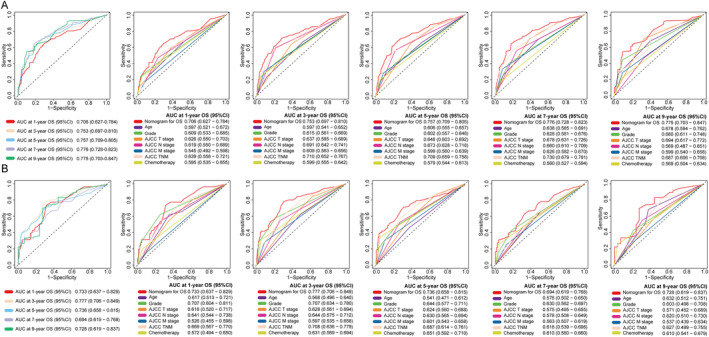
Time‐dependent ROC curves comparing the prognostic accuracy of the 1‐, 3‐, 5‐, 7‐, 9‐year OS prediction model with clinical risk factors in the training (A) and testing (B) cohort. Receiver operating characteristic (ROC) curves of the nomogram of prognosis of NF‐Pan‐NET patients; the AUC values of the ROC curve predicted prognosis of NF‐Pan‐NET patients for the nomogram in the training cohort and testing cohort.

**FIGURE 5:**
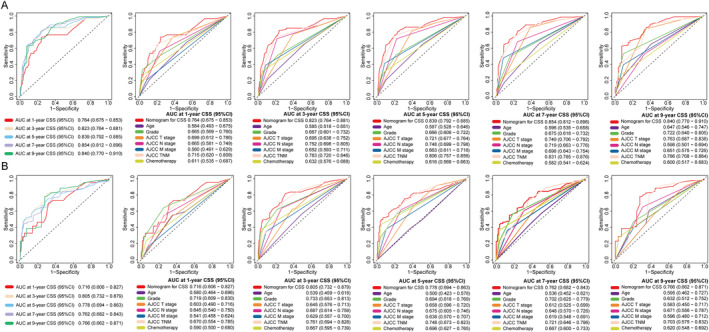
Time‐dependent ROC curves comparing the prognostic accuracy of the 1‐, 3‐, 5‐, 7‐, 9‐year CSS prediction model with clinical risk factors in the training (A) and testing (B) cohorts. Receiver operating characteristic (ROC) curves of the nomogram of prognosis of NF‐Pan‐NET patients; the AUC values of the ROC curve predicted prognosis of NF‐Pan‐NET patients for the nomogram in the training cohort and testing cohort. OS: Overall survival. CSS: Cancer‐specific survival.

**FIGURE 6:**
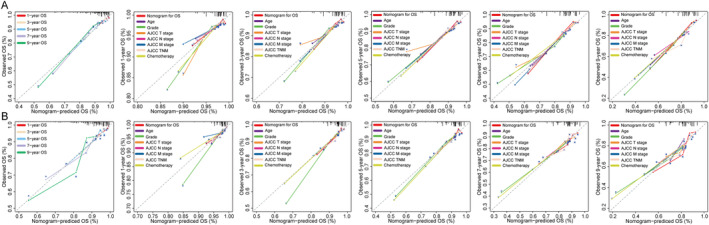
The calibration curves of the nomogram and AJCC stage and other variables of 1‐, 3‐, 5‐, 7‐, 9‐year OS in the training (A) and testing (B) cohorts. OS, overall survival.

**FIGURE 7:**
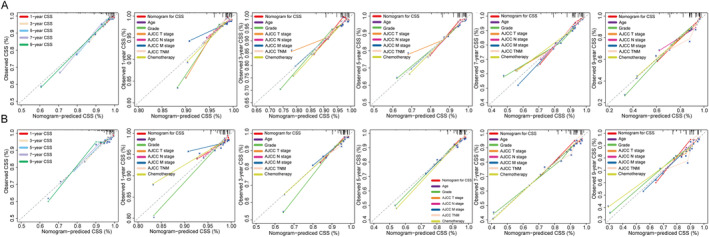
The calibration curves of the nomogram and AJCC stage and other variables of 1‐, 3‐, 5‐, 7‐, 9‐year CSS in the training (A) and testing (B) cohorts. CSS, cancer‐specific survival.

**FIGURE 8:**
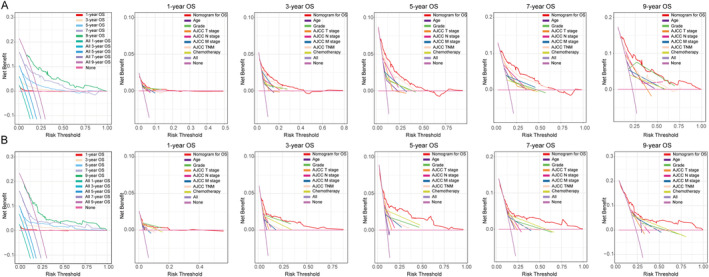
The decision curves of the nomogram of OS prediction model and AJCC stage and other variables in the training (A) and testing (B) cohorts. OS, overall survival.

**FIGURE 9:**
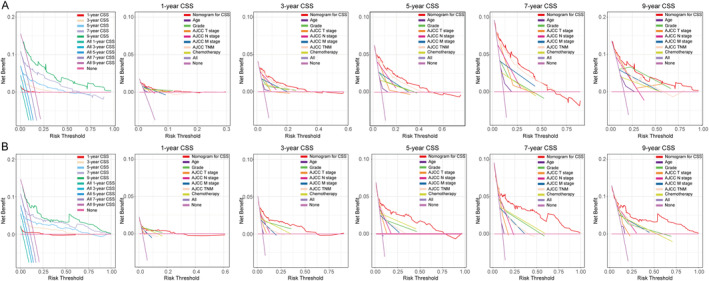
The decision curves of the nomogram of CSS prediction model and AJCC stage and other variables in the training (A) and testing (B) cohorts. CSS, cancer‐specific survival.

C‐index of the nomogram for OS was 0.754 (95% CI: 0.667–0.840) in the training cohort and 0.747 (95% CI: 0.631–0.862) in the testing cohort, while for CSS, it was 0.822 (95% CI: 0.730–0.914) in the training cohort and 0.784 (95% CI: 0.642–0.925) in the testing cohort. Furthermore, compared to other single‐variable models and the AJCC 8th TNM staging system, the nomograms demonstrated higher accuracy in predicting OS and CSS overall (Figures 4 and 5). In addition, time‐dependent ROC and C‐index curves consistently confirmed that the nomograms outperformed the AJCC 8th TNM staging system for prognostic evaluation at any given point‐in‐time (Figure S1). Calibration curves were developed to assess model performance as shown in Figures 6 and 7. A closer alignment between solid model line and diagonal light gray line indicates more accurate survival prediction by the model. The calibration curves revealed a good consistency across different prognostic points‐in‐time, even up to a 9‐year survival prediction, which significantly surpassed performance of AJCC TNM staging system. Moreover, DCA and CIC curves indicated that the net clinical benefit of the nomograms exceeded the single‐variables model and the AJCC 8th TNM staging system when predicting survival for NF‐Pan‐NET patients with pancreatectomy which would be more effective than a treat‐none or treat‐all strategy (Figures 8 and 9 and Figure S2). Taken together, these results highlight outstanding predictive performance of our nomograms for OS and CSS among NF‐Pan‐NET patients after pancreatectomy.

### 3.4 Novel Risk Stratification Based on the Nomograms

**FIGURE 10:**
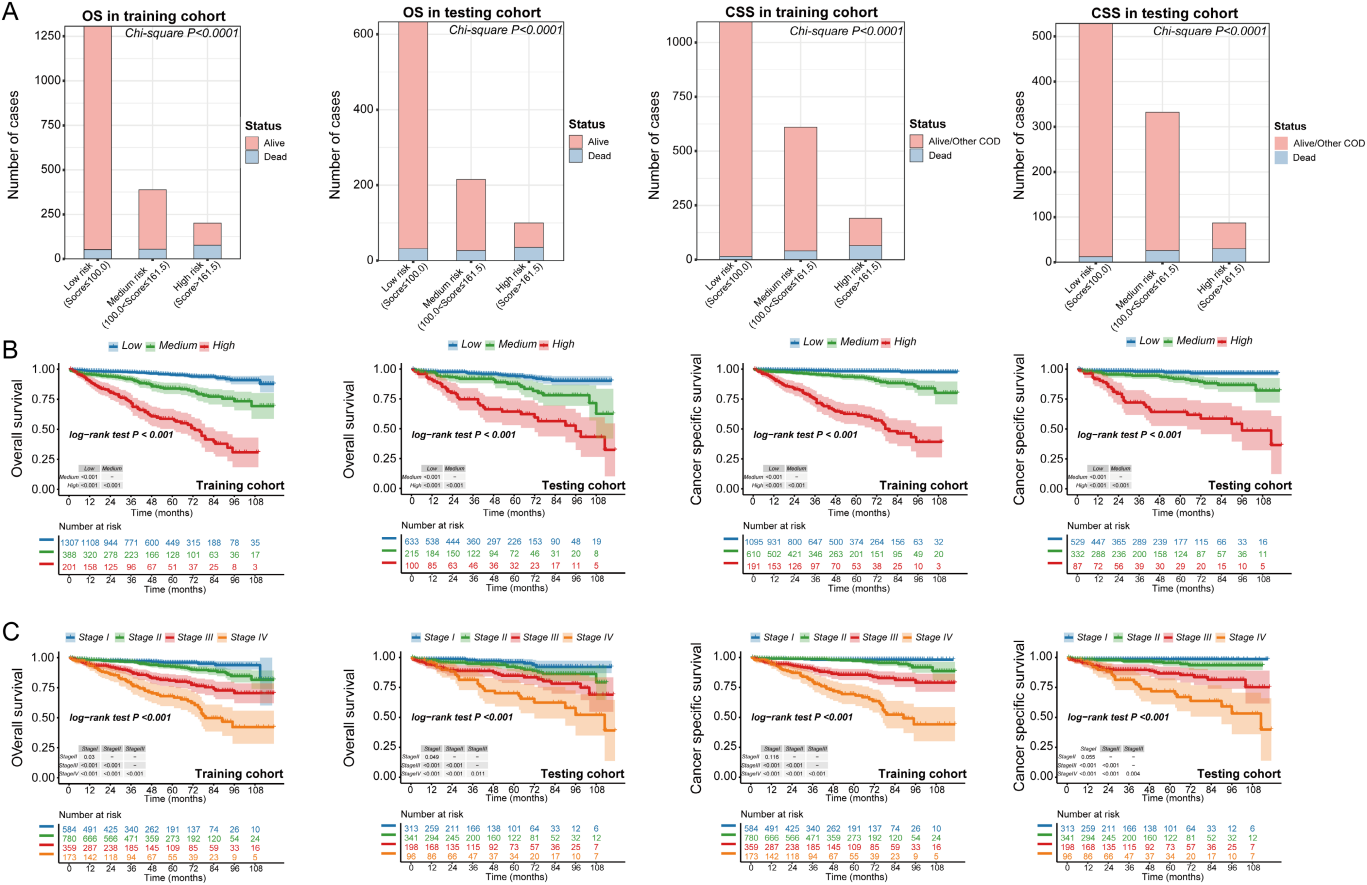
The OS and CSS status of NF‐Pan‐NET patients after pancreatectomy in the low‐risk and high‐risk groups between training and testing cohorts (A). Kaplan–Meier curves for OS and CSS of NF‐Pan‐NET patients after pancreatectomy in the low‐, medium‐ and high‐risk groups between training and testing cohorts (B). Kaplan–Meier curves for OS and CSS of NF‐Pan‐NET patients after pancreatectomy in different AJCC 8th TNM stages between training and testing cohorts (C).

Given the commendable performance of the prognostic nomograms, the total scores of the patients were calculated based on the nomograms to establish a risk stratification system for both OS and CSS. X‐tile software was employed to determine the optimal cutoff values for the total points (Figure S1). Based on these cutoff values, all patients were categorized into three risk subgroups: low risk (total score ≤ 100, *n* = 1940), medium risk (100 < total score ≤ 161.5, *n* = 603) and high risk (total score > 161.5, *n* = 301) for OS and low risk (total score ≤ 100, *n* = 1624), medium risk (100 < total score ≤ 251, *n* = 942) and high risk (total score > 251, *n* = 278) for CSS. Notably, compared to patients in the low‐risk and medium‐risk groups, those in the high‐risk group exhibited significantly higher mortality rates (Chi‐square *p* < 0.0001) (Figure 10A). In Kaplan–Meier curves, significant differences in survival among the three risk subgroups in both training and testing cohorts were revealed, thereby confirming the efficacy of this novel risk stratification system (Figure 10B). Furthermore, this novel system effectively resolved the issue of indistinguishable survival between AJCC 8th TNM Stage I and Stage II (Figure 10C).

## 4 Discussion

The prognostic evaluation and postoperative follow‐up strategy in NF‐Pan‐NET patients after pancreatectomy have been extensively discussed, representing a focal issue for both patients and clinicians. In the present study, we firstly constructed nomograms and risk stratification systems for predicting both OS and CSS using the same six clinicopathological variables and treatment regimens. During the development of these nomograms, we aimed to simplify the included variables as much as possible while ensuring their significance in both OS and CSS, resulting in user‐friendly models suitable for clinical application. Unlike pancreatic ductal adenocarcinoma, NF‐Pan‐NET patients exhibit significantly longer survival times, particularly those who received pancreatectomy. Therefore, our nomograms included long‐term survival rates such as 9‐year survival rate. Various statistical methods were employed to validate their excellent performance in predicting prognosis. The prognostic nomograms and risk stratification systems can facilitate clinical prognostic evaluation and individualized follow‐up.

The number of surgical resections for NF‐Pan‐NET has been increasing recently due to the rising morbidity rates and advancements in surgical techniques [17, 18]. During the data screening process of our study, almost half of the NF‐Pan‐NET patients underwent pancreatectomy, and this proportion may even exceed half in recent years. However, there is still ongoing controversy regarding the optimal management of small NF‐Pan‐NETs (≤2 cm). A recent study suggested that certain conditions such as younger diagnostic age (<65 years), absence of comorbidities, and distal pancreatic tumors could provide survival benefits for patients with small NF‐Pan‐NETs after pancreatectomy [19]. Moreover, surgical resection may be necessary for small NF‐Pan‐NETs exhibiting malignant signs like lymph node metastasis, G2–3 grading, and microvascular invasion [20]. In addition, surgical resection has also been shown to prolong survival in G3 NF‐Pan‐NETs, particularly when Ki‐67 <55% [21].

Given the escalating number of patients with NF‐Pan‐NET after pancreatectomy, it is imperative to develop an effective prognostic system that can guide clinical treatment and personalized postoperative follow‐up. Although the AJCC TNM staging system is recognized for its ability to predict cancer prognosis, certain limitations still persist. The AJCC TNM staging system solely relies on three clinicopathological variables and fails to incorporate important baseline characteristics such as age at diagnosis, gender, marital status, and significant pathological features like tumor grade and microvascular invasion. Furthermore, it overlooks other therapeutic modalities including surgery, chemotherapy, and radiotherapy. Therefore, incorporation of AJCC TNM staging system with other variables may overcome the shortcoming of AJCC TNM staging system in prognostic prediction. A previous study demonstrated that nomograms integrating AJCC 7th and 8th TNM classifications with age at diagnosis, primary site, grade, and primary site surgery exhibited an advantage over the AJCC TNM staging system itself [22]. Besides, a study of Song et al. also indicated nomograms incorporating age, grade, and surgical treatment with the AJCC TNM staging system optimized prognostic prediction efficacy [23].

In our study, we developed novel nomograms incorporating AJCC T stage, AJCC N stage, AJCC M stage, age at diagnosis, grade, and chemotherapy to accurately predict OS and CSS in patients with NF‐Pan‐NETs after pancreatectomy. Age at diagnosis and grade have consistently emerged as crucial prognostic factors for NF‐Pan‐NET patients [14, 22, 23]. To enhance prognostic discrimination, we determined optimal cutoff values for age at diagnosis using X‐tile software. Grade of NF‐Pan‐NET is based on Ki‐67 proliferative index and mitotic rate. Ki‐67 proliferation index has been implicated as a risk factor of liver metastasis and local invasion while also being associated with tumor recurrence [24, 25]. Therefore, the result of our study consistent with previous studies. However, it is intriguing to note that chemotherapy emerges as a negative independent prognostic factor for patients with NF‐Pan‐NET after pancreatectomy. The efficacy of chemotherapy in Pan‐NET remains controversial. Chemotherapeutics in NET include platinum/etoposide, temozolomide, and capecitabine (TEMCAP), as well as oxaliplatin. A recent review highlighted that G3 neuroendocrine carcinoma typically receives platinum/etoposide chemotherapy as first‐line treatment, while the use of chemotherapy as initial therapy for G1–2 neuroendocrine tumors is not routine practice. Moreover, evidence supporting adjuvant chemotherapy for resected G1–2 NETs is scarce [26]. Besides, a study implied that chemotherapy could enhance the survival rates for Pan‐NET patients with poorly differentiated and undifferentiated tumor grading [13]. However, a retrospective study indicated that adjuvant cytotoxicity chemotherapy of gastroenteropancreatic neuroendocrine tumors (GEP‐NET) patients after resection did not provide any benefits and was even negatively associated with recurrence‐free survival (RFS) [27]. Besides, another study also indicated no improvement in survival for patients with resected gastric neuroendocrine carcinomas (G‐NECs) or mixed gastric adenoneuroendocrine carcinomas (G‐MANECs) who received fluorouracil‐based chemotherapy [28]. In the present study, a total of 153 patients underwent chemotherapy, with 94 receiving adjuvant chemotherapy and an additional 37 receiving neoadjuvant chemotherapy. Furthermore, it should be noted that even patients with G1–2 tumors were included in the chemotherapy group, which may have contributed to the observed differences in therapeutic regimens. However, it is worth questioning whether adjuvant chemotherapy is truly ineffective for pancreatic neuroendocrine tumors. Lamberti et al. suggested that potential biases exist among the included patients and emphasized the need for rigorous patient selection in future prospective multicentric studies [29].

The result of our study suggested that neither tumor size nor AJCC T stage was not independent prognostic factors in NF‐Pan‐NET patients after pancreatectomy. Previous research by Liu et al. also found that tumor size was not a significant prognostic factor in their multivariate Cox analysis [23]. In addition, a nationwide study conducted in Korea indicated that although tumor size did not directly impact RFS, it served as an indirect indicator due to its strong correlation with grade and lymph node metastasis, thus influencing treatment decisions [30]. In our present study, tumor size was not significant prognostic both in LASSO and multivariate Cox regression. However, considering the high collinearity between AJCC T stage and tumor size, and the fact that AJCC T stage showed significance in LASSO Cox regression for OS and CSS, we included AJCC T stage in constructing the final nomograms for prognostic prediction. Moreover, although a previous study indicated that female gender was a favorable prognostic factor of NF‐Pan‐NET patients after pancreatectomy, the prognosis differences were not significant between genders in our present study [31]. The potential reasons were complicated. The inclusion of patients from different year periods and the follow‐up time may be contributing factors.

Although the nomogram and the risk evaluation systems demonstrated favorable performance, there still some limitations in our study. Firstly, due to a wide time span encompassing the included patients, there were biases in therapeutic regimens that influenced patient survival times. Besides, selection bias may have been present as patients with poor clinicopathological variables who were more likely to receive chemotherapy. Second, external validation based on data from our hospitals was not conducted due to a lack of integrated data. Moreover, certain clinicopathological features such as venous invasion, lymphatic invasion, status of surgical margins, and Ki‐67 could not be obtained in SEER database despite their prognostic value [32]. In addition, some inconsistencies between the analysis results of the training cohort and testing cohort were observed due to inevitable significantly differences between these two cohorts. However, these discrepancies did not significantly impact the overall results. Given the aforementioned limitations, future prospective studies should focus on validating the constructed nomograms and risk stratification systems specifically within Chinese populations.

## 5 Conclusion

In conclusion, we developed, constructed, and validated a novel model based on AJCC 8th TNM staging system to accurately predict OS and CSS in patients with NF‐Pan‐NET after pancreatectomy. Despite inevitable limitations, the nomograms and risk stratification systems can effectively guide clinicians to formulating appropriate follow‐up strategies based on varying risk ratings, thereby demonstrating promising clinical applicability.

## Author Contributions

Conception and design: Yizhi Wang and Yang Kong. Funding support: Dongkai Zhou. Provision of study materials: Yang Kong and Dongkai Zhou. Collection and assembly of data: Yizhi Wang and Qifan Yang. Data analysis and interpretation: Yizhi Wang, Qifan Yang and Dongkai Zhou. Manuscript writing: all authors. Final approval of manuscript: all authors.

## Ethics Statement

This study was granted exemption by the Institutional Review Board of Zhejiang University because of the publicity and anonymity of SEER data.

## Conflicts of Interest

The authors declare no conflicts of interest.

## Supporting information


**File S1**. Supplemenatary Information.

**Figure S1** Identification of the optimal cutoff values for the variables “age,” “tumor size,” “regional nodes examined (RNE),” “year of diagnosis,” “overall survival (OS) scores,” and “cancer‐specific survival (CSS) scores” via X‐tile software analysis. (A–F) Histograms of patient distribution according to the age, tumor size, RNE, year of diagnosis, OS scores, and CSS scores, respectively. (F–J) The Kaplan–Meier curves of the age, tumor size, RNE, year of diagnosis, OS scores, and CSS scores of NF‐Pan‐NET patients after pancreatectomy, respectively.

**Figure S2** Time‐dependent ROC curves comparing the prognostic accuracy of the OS and CSS prediction model with clinical risk factors in the training and testing cohorts (A). Time‐dependent C‐index curves comparing the prognostic accuracy of the OS and CSS prediction model with clinical risk factors in the training and testing cohorts (B).

**Figure S3** CIC curves of the OS and CSS prediction model in the training and testing cohorts (A). CIC curves of the OS and CSS prediction of AJCC 8th TNM staging system in the training and testing cohorts (B).

## Data Availability

The data that support the findings of this study are available from the corresponding author upon reasonable request.
