# Supplementary figures and images for: Prognostic Nomograms for Patients With NF‐Pan‐NET After Pancreatectomy: A Retrospective Analysis Based on SEER Database
Source: Cancer Rep (Hoboken). 2024 Sep 5;7(9):e2165. doi: 10.1002/cnr2.2165 (PMC11375333; doi:10.1002/cnr2.2165)

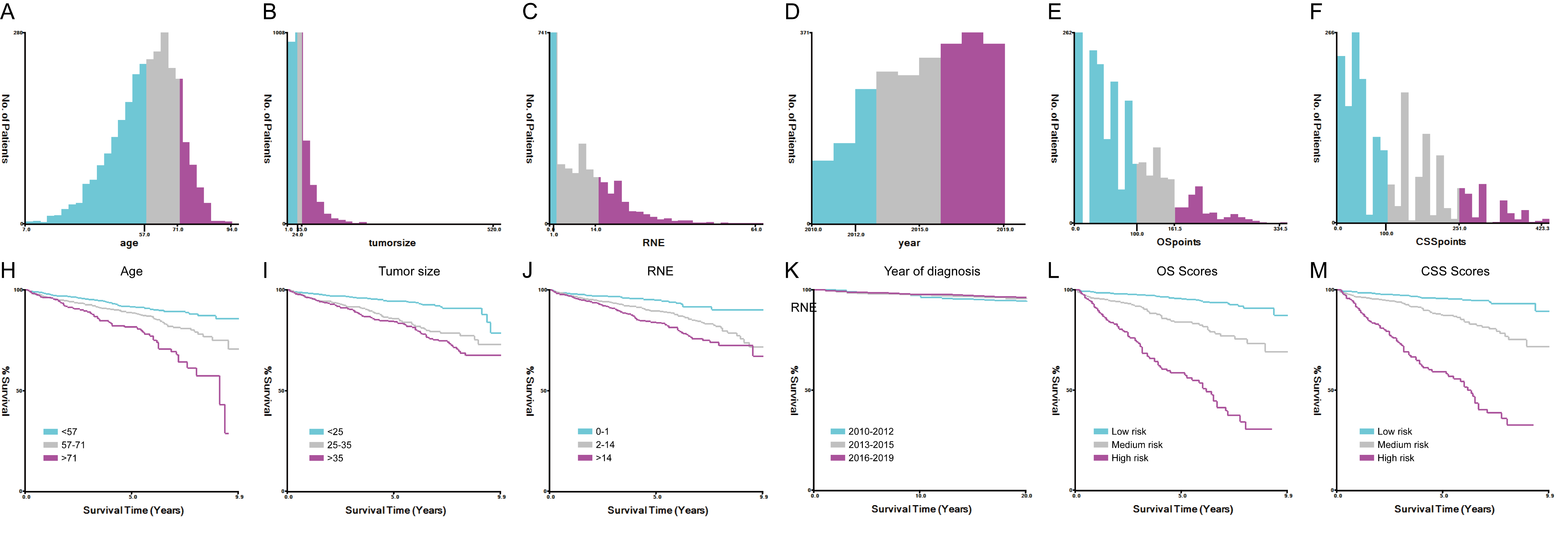

Supplement: Supplementary file 2 — Figure S1 Identification of the optimal cutoff values for the variables “age,” “tumor size,” “regional nodes examined (RNE),” “year of diagnosis,” “overall survival (OS) scores,” and “cancer‐specific survival (CSS) scores” via X‐tile software analysis. (A–F) Histograms of patient distribution according to the age, tumor size, RNE, year of diagnosis, OS scores, and CSS scores, respectively. (F–J) The Kaplan–Meier curves of the age, tumor size, RNE, year of diagnosis, OS scores, and CSS scores of NF‐Pan‐NET patients after pancreatectomy, respectively. [file CNR2-7-e2165-s003.tif]

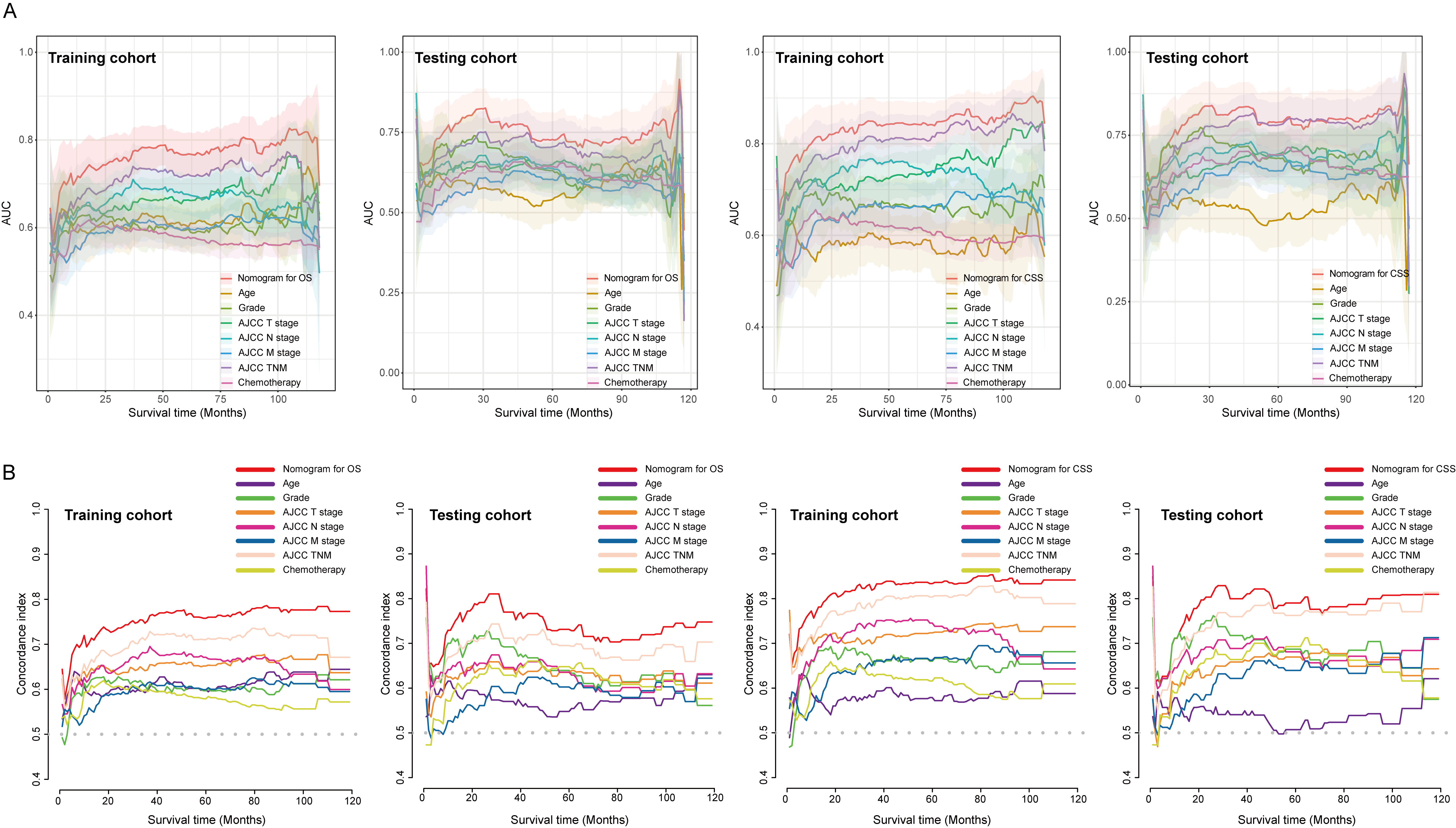

Supplement: Supplementary file 3 — Figure S2 Time‐dependent ROC curves comparing the prognostic accuracy of the OS and CSS prediction model with clinical risk factors in the training and testing cohorts (A). Time‐dependent C‐index curves comparing the prognostic accuracy of the OS and CSS prediction model with clinical risk factors in the training and testing cohorts (B). [file CNR2-7-e2165-s002.tif]

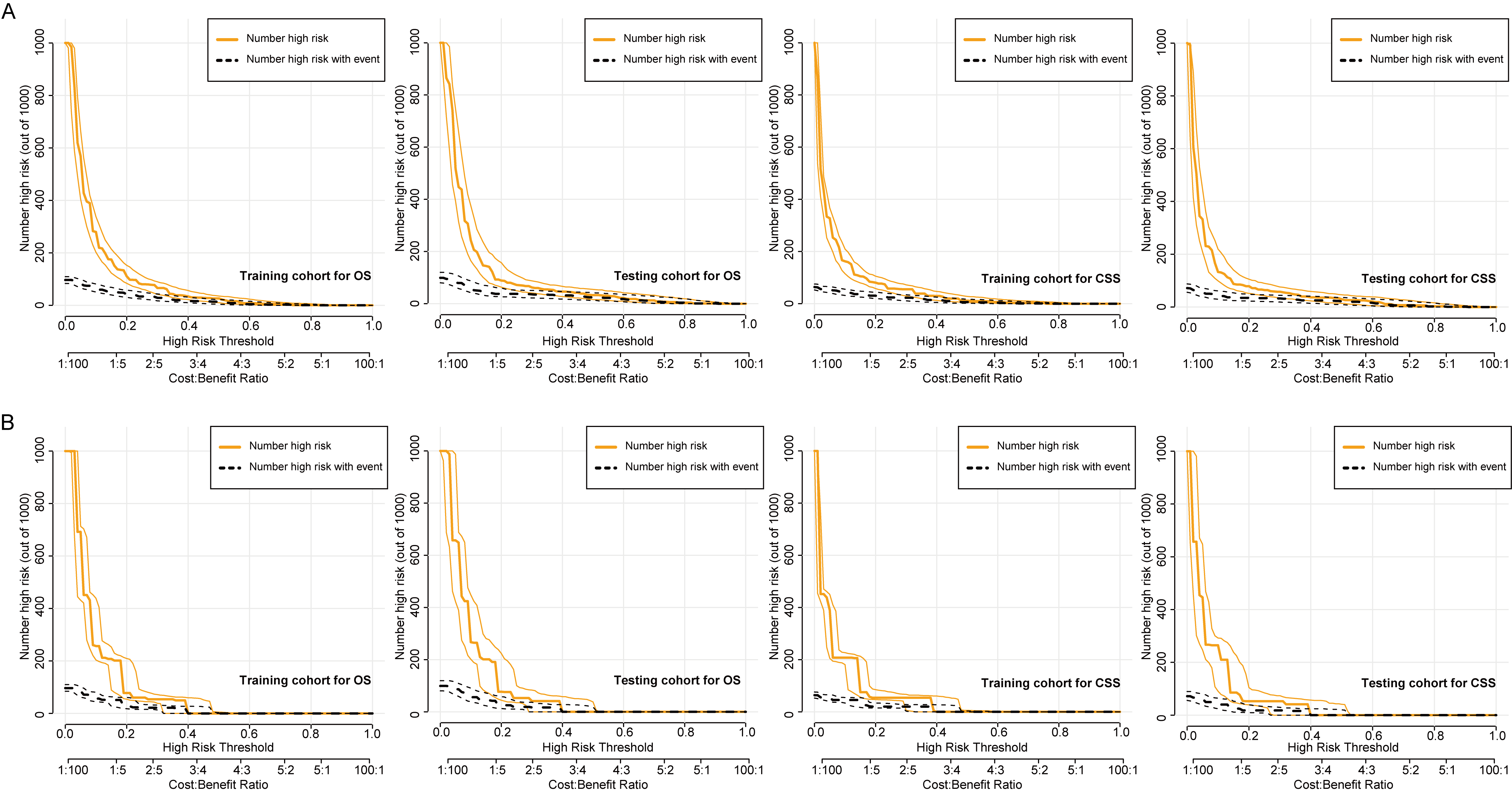

Supplement: Supplementary file 4 — Figure S3 CIC curves of the OS and CSS prediction model in the training and testing cohorts (A). CIC curves of the OS and CSS prediction of AJCC 8th TNM staging system in the training and testing cohorts (B). [file CNR2-7-e2165-s001.tif]
